# Board certification and urban–rural migration of physicians in Japan

**DOI:** 10.1186/s12913-018-3441-y

**Published:** 2018-08-07

**Authors:** Soichi Koike, Masatoshi Matsumoto, Hideaki Kawaguchi, Hiroo Ide, Hidenao Atarashi, Kazuhiko Kotani, Hideo Yasunaga

**Affiliations:** 1 Division of Health Policy and Management, Center for Community Medicine, Jichi Medical University, 3311-1 Yakushiji, Shimotsuke, Tochigi, 329-0498 Japan; 2 Division of Public Health, Center for Community Medicine, Jichi Medical University, 3311-1 Yakushiji, Shimotsuke, Tochigi, 329-0498 Japan; 3 Department of Community Based Medical System, Institute of Biomedical and Health Sciences, Hiroshima University, 1-2-3 Kasumi, Minami-ku, Hiroshima, Hiroshima, 734-8551 Japan; 4 Department of Biomedical Informatics, Graduate School of Medicine, The University of Tokyo, 7-3-1 Hongo, Bunkyo-ku, Tokyo, 113-0033 Japan; 5 Department of Medical Community Network and Discharge, Chiba University Hospital, 1-8-1 Inohana, Chuo-ku, Chiba, Chiba, 260-8677 Japan; 6 Department of Healthcare Information Management, The University of Tokyo Hospitals, The University of Tokyo, 7-3-1 Hongo, Bunkyo-ku, Tokyo, 113-0033 Japan; 7 Division of Community Medicine, Center for Community Medicine, Jichi Medical University, 3311-1 Yakushiji, Shimotsuke, Tochigi, 329-0498 Japan; 8 Department of Clinical Epidemiology and Health Economics, School of Public Health, The University of Tokyo, 7-3-1 Hongo, Bunkyo-ku, Tokyo, 113-0033 Japan

**Keywords:** Board certification, Maintenance of board certification, Urban–rural migration, Physician distribution

## Abstract

**Background:**

The board certification system serves as a quality assurance system for physicians, and its design and operation are important health policy issues. In Japan, board certification was established and operated independently by academic societies and has not been directly linked to reimbursement systems. The phenomenon of younger physicians seeking specialist careers has raised concerns about acceleration of the tendency of fewer physicians working in rural areas and the maldistribution of physicians. Little is known about the associations between physicians’ geographical migration patterns and board certification status changes or between the continuation of urban/rural practice and the maintenance of board certification. This study aimed to identify these associations and to discuss their policy implications.

**Methods:**

We analyzed 2012 and 2014 data from the Survey of Physicians, Dentists, and Pharmacists, a national census survey. To analyze geographical migration patterns, transitions in practice location (rural, intermediate, and urban) were analyzed by board certification status change (new, lost, consistently certified, and consistently uncertified). Logistic regression analysis was conducted to assess whether the odds of migrating to more urban/rural municipalities were associated with board certification status changes, adjusting for covariates, and whether practicing in a rural area was associated with maintaining board certification.

**Results:**

Among 18,726 newly board-certified physicians, 94.9% (13,435/14,150) of those working in urban areas before certification remained in urban areas, whereas 64.6% (393/608) of those working in rural areas stayed in rural areas. Those who were newly certified had higher odds of moving to more urban areas, adjusting for covariates. Those who stayed in rural areas showed lower odds of maintaining board certification, adjusting for covariates.

**Conclusions:**

Newly board-certified physicians are more likely to migrate to other types of areas, particularly more urban areas, than other physicians. Allocating more training quotas to rural areas could be one option for leveling the distribution of specialists. It also appeared that those practicing in rural areas have difficulty maintaining their certification, so the need to establish a support system for already-certified physicians in rural areas should be emphasized.

**Electronic supplementary material:**

The online version of this article (10.1186/s12913-018-3441-y) contains supplementary material, which is available to authorized users.

## Background

Both securing health care quality and achieving a fair distribution of medical resources are important health care policy issues. However, these two goals are sometimes incompatible. As medical science advances, more physicians pursue specialty careers, but most authorities believe that more generalists are required [1]. Board certification systems are quality assurance systems for physicians, and the design and operation of these systems are important. Existing evidence suggests that board certification is correlated with better patient outcomes and better quality of care [2–4].

In Japan, board certification systems were established and operated independently by academic societies. These systems have not been directly linked to reimbursement through the health insurance system. Because each academic society developed its own policy for certification, there has been a lack of coordination and harmonization of the certification criteria and process. The establishment of the Panel on Board Certification within the Ministry of Health, Labour and Welfare (MHLW) was intended to review a wide range of issues around the board certification system, such as how to improve the quality of board-certified specialists and provide better care for patients as well as how to design a new board certification system that does not accelerate the maldistribution of physicians. In 2013, the Panel recommended the establishment of a two-step board certification system (i.e., subspecialty certification can only be obtained after general area board certification) and proposed general practice as a new general area of board certification [5]. Training for board certification under this new policy is scheduled to start in fiscal year 2018. According to the New Maintenance Standard for Board Certification System published by the Japanese Medical Specialty Board in December 2016 [6], qualification for board certification requires at least 5 years of clinical experience after becoming licensed as a physician. In addition to case logs of the candidate’s experience, training records such as clinical safety, infectious disease control, and ethics as well as academic achievements are assessed. After passing the examination for qualification, an achievement assessment is conducted. Certification is valid for 5 years.

Uneven geographical distribution, particularly that related to clinical training and specialty choice is an issue in many countries [7–15]. In Japan as well, younger physicians seeking specialist careers has raised concern that fewer physicians will work in rural areas and that the concentration of physicians in urban areas will increase [16, 17] and this concern has drawn public attention as relative shortages of physicians in rural areas have accelerated [18–20].

Several studies have examined whether working at a hospital in an urban area versus a hospital in a rural area is associated with obtaining and maintaining certification. One study showed that rural family physicians were more likely to maintain their certification [21], whereas another study found that hospital location (urban, suburban, rural or inner city) was not associated with the American Board of Family Medicine’s first-time pass rate [22]. Likewise, in Japan, a previous study showed that the odds of keeping board certification in internal medicine and subspecialty areas were not associated with the municipality type of the practice location (city or town/village) [23]. With regard to urban/rural migration, migration patterns were studied in the United States [24, 25]. However, little is known about the association of physicians’ geographical migration patterns with obtaining or losing board certification or about the association between continuation of urban/rural practice and board certification status. Therefore, the present study aimed to identify the associations between 1) board certification status and geographical migration and 2) practice location and maintenance of certification. We also discuss the policy implications of the findings.

## Methods

The Survey of Physicians, Dentists, and Pharmacists is a national census survey conducted by the MHLW every 2 years. The Medical Practitioners’ Act requires all medical practitioners report their status every 2 years, and the survey questionnaires are completed by the medical practitioners themselves. The response rates for the Survey of Physicians, Dentists, and Pharmacists have not been published by the MHLW but are estimated to be approximately 90% [26].

We used data from the survey with permission from the MHLW. Data on the registration number, year of registration as a physician, sex, workplace type (municipality type and medical institution type), area of practice, and board certification status for each physician in 2012 and 2014 were evaluated in this study. We used the physician registration number to establish a cohort dataset. Board certification status and geographical migration patterns between the two survey periods were then analyzed. The municipality borders that changed because of mergers were adjusted in the two periods. In total, 1896 geographical areas that included all municipalities were identified and used for this study. These areas were classified into three categories based on population density: first tertile (lowest population density, rural), second tertile (second-lowest density, intermediate), and third tertile (highest density, urban). The cut off points of population density were 106.0/km2 and 629.8/km^2^. As Japan has not rurality criteria similar to the Office of Management and Budget (OMB) standards in the United States [27], we used a classification based on population density. A previous study in Japan [28] employed population density and used quartiles, but the number of segments was arbitrary, and thus three segments were employed in the present study. Moving to a more urban area was defined as working in a rural area in 2012 and moving to an intermediate or urban area in 2014 or working first in an intermediate area and then moving to an urban area. Moving to a less urban area was defined working in an urban area in 2012 and moving to an intermediate or rural area in 2014 or working first in an intermediate area and then moving to a rural area.

To assess urban–rural movement patterns between the two survey periods, 3 × 3 tables (rural, intermediate, and urban in 2012 × rural, intermediate, and urban in 2014) were prepared for each board certification status change from 2012 to 2014: no board certification in either year; board certification in 2014 but not 2012 (newly board certified); board certification in both years (maintained board certification); and board certification in 2012, but not in 2014 (lost board certification).

To assess whether board certification status change was associated with geographical migration, logistic regression analyses were conducted. In each municipality-type tertile, we tested whether the odds of migrating to more urban municipalities were associated with board certification status change from 2012 to 2014, adjusting for sex (male/female), years since registration as a physician (0–14, 15–29, 30–44, or ≥ 45), and type of workplace in 2014 (hospital/clinic or other). Logistic regression analysis was also performed to assess whether the odds of migrating to more rural municipalities were associated with board certification status changes from 2012 to 2014, adjusting for the same covariates. A sub-analysis of the individual specialties was conducted to examine any differences from the total group.

Logistic regression was also used to assess whether practicing in a rural area was associated with maintaining board certification status, testing whether the odds of holding board certification was associated with practice location: staying in a rural area (working in rural municipalities in both 2012 and 2014), staying in an urban area (working in urban municipalities in both 2012 and 2014), and others, adjusting for sex, years since registration as a physician, and type of institution in 2012. A sub-analysis of the individual specialties was conducted to examine any differences from the total group.

In these analyses, we defined board-certified physicians as physicians with board certification in general internal medicine, surgery, pediatrics, obstetrics and gynecology, orthopedics, neurosurgery, ophthalmology, otorhinolaryngology, acute medicine, anesthesiology, dermatology, urology, plastic surgery, radiology, pathology, or rehabilitation. In Japan, the abovementioned 16 areas and psychiatry, laboratory medicine, and general practice have been defined as general areas of board certification. However, we were unable to include the latter three areas in the analysis because insufficient data were available from the Survey of Physicians, Dentists, and Pharmacists and it was not possible to calculate status changes for these area from 2012 to 2014. Data on board certification status in psychiatry was not collected in the 2012 survey, and data on board certification status in laboratory medicine was not collected in either survey. Board certification in general practice began after the study period. There were 8293 physicians board certified in psychiatry in 2014 [29], and 588 physicians board certified in laboratory medicine in August 2016 [30].

Population density was calculated based on the basic resident population register as of 01 January, 2015, by the Ministry of Internal Affairs and Communications [31], and municipality size was based on the Statistical Reports on the Land Area by Prefectures and Municipalities in Japan by the Geospatial Information Authority [32]. In this analysis, 2015 population data were applied to municipalities for both 2012 and 2014 to set the urban–rural classification of the municipalities for the study period. We used the population data in 2015, because the 2014 survey was conducted on 31 December 2014.

For the statistical analyses, *P*-values less than 0.05 were considered significant. SPSS Version 22.0 J software (Japan IBM, Tokyo, Japan) was used for all statistical analyses.

## Results

A total of 282,308 physicians complted both the 2012 and 2014 surveys. Of these respondents, 115,898 (41.1%) in 2012 and 123,822 (43.9%) in 2014 were board certified in at least one general area of board certification (Table 1).

**Table 1:** Characteristics of the study subjects (*n* = 282,308)

	2012 Survey		2014 Survey	
Sex, n, %				
Male	228,024	80.8	228,024	80.8
Female	54,284	19.2	54,284	19.2
Years of experience, n, %				
0–14	97,825	34.7	85,298	30.2
15–29	104,459	37.0	102,904	36.5
30–44	60,221	21.3	67,965	24.1
≥ 45	19,803	7.0	26,141	9.3
Workplace, n, %				
Tertile 1 (Rural)	11,151	3.9	11,168	4.0
Tertile 2 (Intermediate)	62,421	22.1	62,064	22.0
Tertile 3 (Urban)	208,736	73.9	209,076	74.1
Institution type, n, %				
Hospital	178,916	63.4	173,779	61.6
Clinic or other	103,392	36.6	108,529	38.4
Area of board certification, n, %				
Any general area of board certification	115,898	41.1	123,822	43.9
General internal medicine	14,142	5.0	15,668	5.5
Dermatology	4787	1.7	5049	1.8
Pediatrics	11,656	4.1	12,347	4.4
Surgery	19,400	6.9	20,171	7.1
Urology	5349	1.9	5648	2.0
Neurosurgery	5993	2.1	6341	2.2
Orthopedics	14,206	5.0	15,048	5.3
Plastic surgery	1666	0.6	1912	0.7
Ophthalmology	8720	3.1	8980	3.2
Otorhinolaryngology	6939	2.5	7212	2.6
Obstetrics and gynecology	9545	3.4	10,223	3.6
Rehabilitation	2342	0.8	2359	0.8
Radiology	4880	1.7	5306	1.9
Anesthesiology	5766	2.0	6415	2.3
Pathology	1676	0.6	1826	0.6
Acute medicine	2834	1.0	3264	1.2
Psychiatry	n.a.		8331	3.0
Laboratory medicine	n.a.		n.a.	
General practice	n.a.		n.a.	

**Fig. 1:**
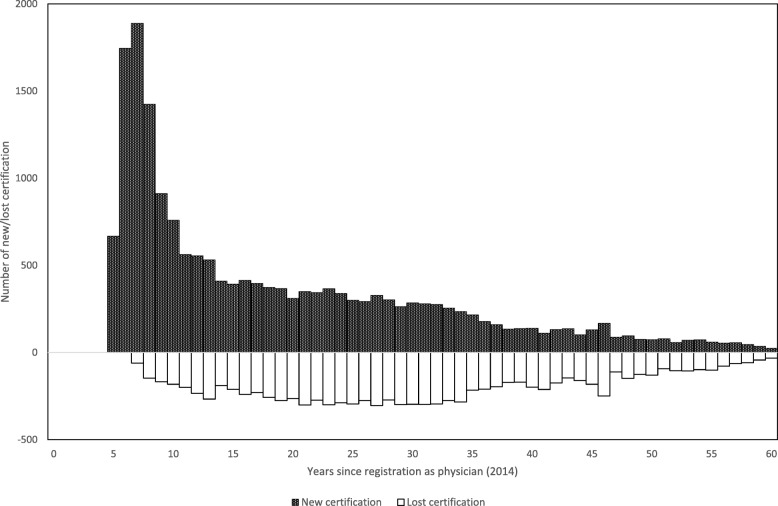
Dynamics of new/lost board certification from 2012 to 2014. The numbers of newly board-certified physicians (black bars) and physicians losing their board certification (white bars) from 2012 to 2014 are presented by years of experience since registration as a physician as of 2014. New board certification peaked at 1888 in the 7th year and gradually declined with further experience. Loss of certification peaked at 304 in the 27th year but showed a gentler slope than that for new certification

The number of physicians who were not board certified in 2012 but were certified in 2014 (newly certified) peaked at the 7th year after registration as a physician and then gradually declined. The number of physicians who lost their general area certification gradually increased, reaching approximately 300 in the 27th year but showed a gentler slope than that for new certification (Fig. 1).

Of those who worked in rural municipalities (tertile 1), 16.9% moved to more urban municipalities—7.5% to intermediate areas (tertile 2) and 9.3% to urban areas (tertile 3)—in 2014. Of those who worked in intermediate areas in 2012, 11.2% moved to more urban areas and 1.4% to less urban area in 2014. Of those who worked in urban areas in 2012, 3.7% moved to less urban areas (urban to intermediate = 3.2%; urban to rural = 0.5%). Among those who were not certified in 2012 but were certified in 2014 (newly certified) and of those who worked in rural area, 35.4% moved to more urban areas (rural to intermediate = 11.8%; rural to urban = 23.5%). Of those who worked in intermediate area 21.9% moved to more urban area and 1.8% moved to less urban area. Of those who worked in urban area, 4.2% moved to less urban area (urban to intermediate = 4.3%; urban to rural = 0.8%) (Table 2).

**Table 2:** Urban–rural migration of physicians from 2012 to 2014

		Municipality type in 2014			
		Tertile 1 (rural)	Tertile 2 (intermediate)	Tertile 3 (urban)	Total
A Total (n = 282,308)					
Municipality type in 2012	Tertile 1 (rural)	9270	839	1042	11,151
		83.1%	7.5%	9.3%	100.0%
	Tertile 2 (intermediate)	856	54,581	6984	62,421
		1.4%	87.4%	11.2%	100.0%
	Tertile 3 (urban)	1042	6644	201,050	208,736
		0.5%	3.2%	96.3%	100.0%
	Total	11,168	62,064	209,076	282,308
		4.0%	22.0%	74.1%	100.0%
B Newly board certified (*n* = 18,726)					
Municipality type in 2012	Tertile 1 (rural)	393	72	143	608
		64.6%	11.8%	23.5%	100.0%
	Tertile 2 (intermediate)	72	3026	870	3968
		1.8%	76.3%	21.9%	100.0%
	Tertile 3 (urban)	107	608	13,435	14,150
		0.8%	4.3%	94.9%	100.0%
	Total	572	3706	14,448	18,726
		3.1%	19.8%	77.2%	100.0%
C Remaining board certified (*n* = 105,096)					
Municipality type in 2012	Tertile 1 (rural)	2856	195	212	3263
		87.5%	6.0%	6.5%	100.0%
	Tertile 2 (intermediate)	172	20,978	1711	22,861
		0.8%	91.8%	7.5%	100.0%
	Tertile 3 (urban)	209	1685	77,078	78,972
		0.3%	2.1%	97.6%	100.0%
	Total	3237	22,858	79,001	105,096
		3.1%	21.7%	75.2%	100.0%
D Lost board certification (n = 10,802)					
Municipality type in 2012	Tertile 1 (rural)	359	28	32	419
		85.7%	6.7%	7.6%	100.0%
	Tertile 2 (intermediate)	39	2114	217	2370
		1.6%	89.2%	9.2%	100.0%
	Tertile 3 (urban)	49	249	7715	8013
		0.6%	3.1%	96.3%	100.0%
	Total	447	2391	7964	10,802
		4.1%	22.1%	73.7%	100.0%
E Remaining without board certification (*n* = 147,684)					
Municipality type in 2012	Tertile 1 (rural)	5662	544	655	6861
		82.5%	7.9%	9.5%	100.0%
	Tertile 2 (intermediate)	573	28,463	4186	33,222
		1.7%	85.7%	12.6%	100.0%
	Tertile 3 (urban)	677	4102	102,822	107,601
		0.6%	3.8%	95.6%	100.0%
	Total	6912	33,109	107,663	147,684
		4.0%	22.0%	74.1%	100.0%

The odds of migrating to a more urban area were higher for those who were newly board certified than for those remaining board certified, after adjusting for sex, years of experience as a physician, and type of institutional workplace (Table 3). The sub-analysis of the individual specialties is shown in Additional file 1. The odds of migrating to a more rural area were higher for those who lost their board certification than for those who remained certified (Table 4). The sub-analysis of the individual specialties is shown in Additional file 2.

**Table 3:** Odds of migrating to *more urban* areas for those working in rural and intermediate municipalities

	Tertile 1 (rural) in 2012 and Tertile 2 (intermediate) or 3 (urban) in 2014		Tertile 2 (intermediate) in 2012 and Tertile 3 (urban) in 2014	
	OR (95% CI)	*P*-value	OR (95% CI)	*P*-value
Sex				
Male	Reference		Reference	
Female	0.89 (0.75–1.06)	0.18	0.92 (0.86–0.98)	0.01
Years of experience				
0–14	Reference		Reference	
15–29	0.13 (0.11–0.14)	< 0.001	0.18 (0.17–0.20)	< 0.001
30–44	0.06 (0.05–0.07)	< 0.001	0.08 (0.07–0.09)	< 0.001
≥ 45	0.05 (0.04–0.07)	< 0.001	0.06 (0.05–0.07)	< 0.001
Type of institution				
Clinic	Reference		Reference	
Hospital	1.86 (1.61–2.13)	< 0.001	1.41 (1.31–1.53)	< 0.001
Board certification status from 2012 to 2014				
Remained certified	Reference		Reference	
Lost certification	1.67 (1.20–2.31)	0.02	1.78 (1.52–2.08)	< 0.001
Newly certified	2.36 (1.87–2.98)	< 0.001	1.86 (1.69–2.06)	< 0.001
Remained uncertified	1.14 (0.99–1.31)	0.06	1.25 (1.17–1.33)	< 0.001

**Table 4:** Odds of migrating to *more rural area* for those working in intermediate and urban municipalities

	Tertile 2 (intermediate) in 2012 and Tertile 1 (rural) in 2014		Tertile 3 (urban) in 2012 and Tertile 2 (intermediate) or 1 (rural) in 2014	
	OR (95% CI)	*P*-value	OR (95% CI)	*P*-value
Sex				
Male	Reference		Reference	
Female	0.55 (0.45–0.68)	< 0.001	0.65 (0.62–0.69)	< 0.001
Years of experience				
0–14	Reference		Reference	
15–29	0.30 (0.25–0.36)	< 0.001	0.34 (0.32–0.36)	< 0.001
30–44	0.21 (0.16–0.26)	< 0.001	0.22 (0.20–0.24)	< 0.001
≥ 45	0.20 (0.14–0.28)	< 0.001	0.15 (0.12–0.17)	< 0.001
Type of institution				
Clinic	Reference		Reference	
Hospital	1.59 (1.31–1.94)	< 0.001	1.99 (1.85–2.14)	< 0.001
Board certification status from 2012 to 2014				
Remained certified	Reference		Reference	
Lost certification	2.72 (1.91–3.88)	< 0.001	1.92 (1.70–2.18)	< 0.001
Newly certified	1.48 (1.12–1.97)	0.006	1.32 (1.21–1.45)	< 0.001
Remained uncertified	1.81 (1.51–2.16)	< 0.001	1.41 (1.34–1.50)	< 0.001

The odds of maintaining board certification were lower for those who practiced in rural areas in both 2012 and 2014 than for those who practiced in urban areas in both 2012 and 2014, after adjusting for sex, years of experiences as a physician, and type of institution (Table 5). The sub-analysis of the individual specialties is shown in Additional file 3.

**Table 5:** Association between practice location and keeping board certification

	OR (95% CI)	*P*-value
Sex		
Male	1.00 (reference)	
Female	1.03 (0.98–1.09)	0.24
Years of experience		
0–14	1.00 (reference)	
15–29	1.08 (1.02–1.15)	0.02
30–44	0.76 (0.71–0.81)	< 0.001
≥ 45	0.27 (0.25–0.29)	< 0.001
Type of institution		
Clinic	1.00 (reference)	
Hospital	1.24 (1.19–1.30)	< 0.001
Practice Location		
Urban practice	1.00 (reference)	
Rural practice	0.85 (0.76–0.96)	0.007
Other	0.93 (0.89–0.97)	0.002

## Discussion

Our analysis has shown that those who were newly board certified tended to migrate to more urban areas more frequently than those with other board certification status changes. Those who practiced in rural areas had lower odds of maintaining a general area of board certification than those practicing in urban areas.

There are two implications of these results. One is that new board certification is one of the best times to intervene to achieve a more equal distribution of specialists. In June 2016, a government panel published an interim report recommending setting of training quotas for specialists in certain areas as a possible policy option [33]. As we shown in the present study, the time just after attaining a new certification is the most mobile period in a physician’s career, and thus setting quotas for training specialists in each region could be an option for achieving a more equitable distribution of specialists. However, whether newly certified physicians move to more rural places than where they trained or stay in urban area had not been sufficiently analyzed. In addition, to achieve more equal distribution of specialist, the allocation of training spots need to be discussed with consideration of physician migration after they completed the training as well. In Japan, initial postgraduate clinical training places have been limited to 110% of the graduate cohort to avoid further concentration of physicians in urban areas, but there are no such strict control measures for specialist training. For postgraduate clinical training, quotas have been set for each prefecture; similarly setting a quota for each prefecture for those seeking specialist training could be a policy option.

Our study showed that practicing in rural areas was associated with a lower odds of maintaining a general area of board certification. Other studies found that rural hospital physicians had less social support [34] and that physicians in their 20s felt that specialist training was one of the main reasons to avoid practicing outside of metropolitan areas and other large cities [35]. Furthermore, a survey on rural physicians showed that accumulation of the necessary clinical experience including operations and other invasive clinical procedures, was one of the biggest hurdles to complete specialist training and that experience gained by regular returns to their training facility to receive guidance from a supervisor, while practicing in a rural area was necessary [36]. These findings reiterate the importance of continuous education and continuous support for those working in rural areas.

These two policy options should be considered and implemented together. Under the current licensing and registration for the insurance system, physicians can freely move to other places as they wish. This may cause reallocation of quotas alone to bring about migration of specialists from rural to urban areas, which would neutralize the policy effect. However, merely retaining specialists in rural areas is not sufficient when a shortage already exists in these areas.

There are some limitations to this study. First, the Survey of Physicians, Dentists, and Pharmacists did not cover all general areas of board certification. The role and function of the current board certification system in general internal medicine will not be compatible with board certification in internal medicine. In this transitional period of the board certification system in Japan, the status of being a Board Certified Member of the Japanese Society of Internal Medicine is a prerequisite for obtaining board certification in subspecialty areas within internal medicine. However, this status was not regarded as a general area of board certification, and the Survey of Physicians, Dentists, and Pharmacists did not collect data on it. There were 83,308 board-certified members as of October 2016 [37]. Second, this study was only able to test for association, so causality was not proven. Previous studies have shown that cost, time, and lack of relevance to current practice are the main reasons for not maintaining or renewing certification in internal medicine [38] and pediatrics [39]. Further studies are required to establish causality. Third, we classified municipality into three types based on population density, but the classification criteria could bring about different interpretation of the results. We think it is necessary to develop a rurality classification for the purpose of health service research. Fourth, because this was a secondary data analysis, there may be other factors associated with the choice of practice location that were not collected in the survey. Whether geographical migration is a cause for or a result of maintaining board certification is still unknown.

## Conclusions

Newly board-certified physicians in Japan are more likely to migrate to places that are different from those where they trained or initially practiced and those tend to be more urban that their prior location. A certain proportion of newly certified physicians also moved to less urban areas. For specialists who are already certified, it appears that those practicing in rural areas have difficulty maintaining their certification. Therefore, to intervene in the distribution of specialists, allocating more training quotas to rural areas to increase newly certified specialists and establishing a support system for physicians in rural areas who are already certified should be emphasized to balance the distribution of specialists.

## Additional files

Additional file 1: Odds of migrating to more urban areas for newly certified physicians working in first tertile areas in 2012 (sub-analysis of the individual specialties). (PDF 50 kb)

Additional file 2: Odds of migrating to more rural areas for newly certified physicians working in second tertile areas in 2012 (sub-analysis of the individual specialties). (PDF 49 kb)

Additional file 3: Rural practice and odds of keeping board certification (sub-analysis of the individual specialties). (PDF 46 kb)

## Abbreviations

CI: Confidence interval
MHLW: Ministry of Health, Labour and Welfare
n.a: Not available
OR: Odds ratio

## References

[1] Cassel CK, Reuben DB (2011). Specialization, subspecialization, and subsubspecialization in internal medicine. N Engl J Med.

[2] Sharp LK, Bashook PG, Lipsky MS, Horowitz SD, Miller SH (2002). Specialty board certification and clinical outcomes: the missing link. Acad Med.

[3] Sutherland K, Leatherman S (2006). Does certification improve medical standards?. BMJ.

[4] Brennan TA, Horwitz RI, Duffy FD, Cassel CK, Goode LD, Lipner RS (2004). The role of physician specialty board certification status in the quality movement. JAMA.

[5] Ministry of Health, Labour and Welfare. Final report of the Panel on Board Certification, April 22, 2013. http://www.mhlw.go.jp/stf/shingi/2r985200000300ju.html. Accessed 11 June 2018.

[6] Japanese Medical Specialty Board. New maintenance standard for board certification system. December 2016. http://www.japan-senmon-i.jp/news/doc/sinseibisisin2016.12.16.pdf. Accessed 11 June 2018.

[7] WHO. Increasing access to health workers in remote and rural areas through improved retention: global policy recommendations. Geneva; 2010. 23741785

[8] Fowkes VK, Campeau P, Wilson SR (1991). The evolution and impact of the national AHEC program over two decades. Acad Med.

[9] Leu HI, Chang WT, Lin MH, Chen TJ, Hwang SJ, Chou LF, Jeng MJ (2017). Urban-rural disparity in geographical and temporal availability of pediatric clinics: a nationwide survey in Taiwan. Pediatr Neonatol.

[10] Siega-Sur JL, Woolley T, Ross SJ, Reeve C, Neusy AJ (2017). The impact of socially-accountable, community-engaged medical education on graduates in the Central Philippines: implications for the global rural medical workforce. Med Teach.

[11] Wurie HR, Samai M, Witter S (2016). Retention of health workers in rural Sierra Leone: findings from life histories. Hum Resour Health.

[12] Sapkota BP, Amatya A (2015). What factors influence the choice of urban or rural location for future practice of Nepalese medical students?. A cross-sectional descriptive study Hum Resour Health.

[13] Pagaiya N, Kongkam L, Sriratana S (2015). Rural retention of doctors graduating from the rural medical education project to increase rural doctors in Thailand: a cohort study. Hum Resour Health.

[14] Rosenblatt RA, Hart LG. *Chapter 3:* physicians and rural America. In: Ricketts TC editor. Rural health in the United States. New York: Oxford University Press; 1999, p38–51.

[15] Brooks RG, Walsh M, Mardon RE, Lewis M, Clawson A (2002). The roles of nature and nurture in the recruitment and retention of primary care physicians in rural areas: a review of the literature. Acad Med.

[16] Sasaki H, Otsubo T, Imanaka Y (2013). Widening disparity in the geographic distribution of pediatricians in Japan. Hum Resour Health.

[17] Japan Medical Association, Japan Hospital Association, All Japan Hospital Association, Association of Japanese Healthcare Corporations, Japanese Association of Psychiatric Hospitals. Joint statement: concerns for new board certification system. June 7, 2016. https://www.med.or.jp/nichiionline/article/004478.html. Accessed 13 Aug 2017.

[18] Kobayashi Y, Takaki H (1992). Geographic distribution of physicians in Japan. Lancet.

[19] Tanihara S, Kobayashi Y, Une H, Kawachi I (2011). Urbanization and physician maldistribution: a longitudinal study in Japan. BMC Health Serv Res.

[20] Inoue K, Matsumoto M, Toyokawa S, Kobayashi Y (2009). Transition of physician distribution (1980–2002) in Japan and factors predicting future rural practice. Rural Remote Health.

[21] Schulte BM, Mannino DM, Royal KD, Brown SL, Peterson LE, Puffer JC (2014). Community size and organization of practice predict family physician recertification success. J Am Board Fam Med.

[22] Mims LD, Mainous AG, Chirina S, Carek PJ (2014). Are specific residency program characteristics associated with the pass rate of graduates on the ABFM certification examination?. Fam Med.

[23] Koike S, Matsumoto M, Ide H, Kawaguchi H, Shimpo M, Yasunaga H (2017). Internal medicine board certification and career pathways in Japan. BMC Med Educ.

[24] Vanasse A, Ricketts TC, Courteau J (2007). Orzanco, MG Randolph R. Asghari S Long term regional migration patterns of physicians over the course of their active practice careers Rural Remote Health.

[25] Ricketts TC, Randolph R (2007). Urban-rural flows of physicians. J Rural Health.

[26] Shimada N, Kondo T (2004). Ishi-Shikaishi-Yakuzaishi chosa no kohyo data wo shiyou shita todokede ritsu no suikei [estimation of actual report rates using data from the survey of physicians, dentists, and pharmacists]. Nihon Koshu Eisei Zasshi [Jpn J Public Health].

[27] Ricketts TC, Johnson-Webb KD, Randolph RK, Ricketts TC (1999). *Chapter 1:* populations and places in rural America. Rural health in the United States.

[28] Matsumoto M, Inoue K, Kajii E (2010). Policy implications of a financial incentive programme to retain a physician workforce in underserved Japanese rural areas. Soc Sci Med.

[29] Ministry of Health, Labour and Welfare. Survey of Physicians, Dentists, and Pharmacists 2014. http://www.mhlw.go.jp/toukei/saikin/hw/ishi/14/. Accessed 13 Aug 2017.

[30] Japan Society of Laboratory Medicine. List of board certified clinical laboratory physicians as of Aug 20, 2016. http://jslm.org/recognition/control/index.html. Accessed 11 June 2018.

[31] Ministry of Internal Affairs and Communications. Basic resident register population as of Jan 1, 2014. https://www.e-stat.go.jp/SG1/estat/GL08020103.do?_toGL08020103_&listID=000001154737&disp=Other&requestSender=estat. Accessed 11 June 2018.

[32] The Geospatial Information Authority of Japan. Statistical reports on the land area by prefectures and municipalities in Japan, 2014. http://www.gsi.go.jp/KOKUJYOHO/OLD-MENCHO-title.htm. Accessed 11 June 2018.

[33] Subcommittee of Demand and Supply of Physicians, Panel of Demand and Supply of Medical Workforce, Ministry of Health, Labour and Welfare. Interim Report, June 3, 2016. http://www.mhlw.go.jp/stf/shingi2/0000126444.html. Accessed 13 Aug 2017.

[34] Saijo Y, Chiba S, Yoshioka E, Kawanishi Y, Nakagi Y, Ito T, Sugioka Y, Kitaoka-Higashiguchi K, Yoshida T (2013). Job stress and burnout among urban and rural hospital physicians in Japan. Aust J Rural Health.

[35] Study Group of the Survey of Physicians’ Duty and Working Preference and Health Policy Bureau, Ministry of Health, Labour and Welfare. Survey of physicians’ duty and working preference. 2017. http://www.mhlw.go.jp/stf/shingi2/0000160954.html. Accessed 13 Aug 2017.

[36] Imamichi H, Kojyo T, Kotani K, Maeda T, Tani K, Iguchi S, Sawada T, Morita Y, Kajii E. Difficulties experienced by doctors practicing in rural areas in acquiring specialist qualifications and possible solutions. J Conf Emerg Med Rural Areas Isolated Islands. 2018. In press.

[37] Japanese Society of Internal Medicine. Number of board certified members of the Japanese Society of Internal Medicine and fellows of the Japanese Society of Internal Medicine by prefecture as of Oct 25, 2016. http://www.naika.or.jp/nintei/seido/meibo/pref47/. Accessed 13 Aug 2017.

[38] Lipner RS, Bylsma WH, Arnold GK, Fortna GS, Tooker J, Cassel CK (2006). Who is maintaining certification in internal medicine—and why? A national survey 10 years after initial certification. Ann Intern Med.

[39] Freed GL, Dunham KM, Althouse LA (2008). Characteristics of general and subspecialty pediatricians who choose not to recertify. Pediatrics.

