# Supplementary material for: Board certification and urban–rural migration of physicians in Japan
Source: BMC Health Serv Res. 2018 Aug 7;18:615. doi: 10.1186/s12913-018-3441-y (PMC6081900; doi:10.1186/s12913-018-3441-y)
Supplement: Supplementary file 2 — Odds of migrating to more rural areas for newly certified physicians working in second tertile areas in 2012 (sub-analysis of the individual specialties). (PDF 49 kb) [file 12913_2018_3441_MOESM2_ESM.pdf]

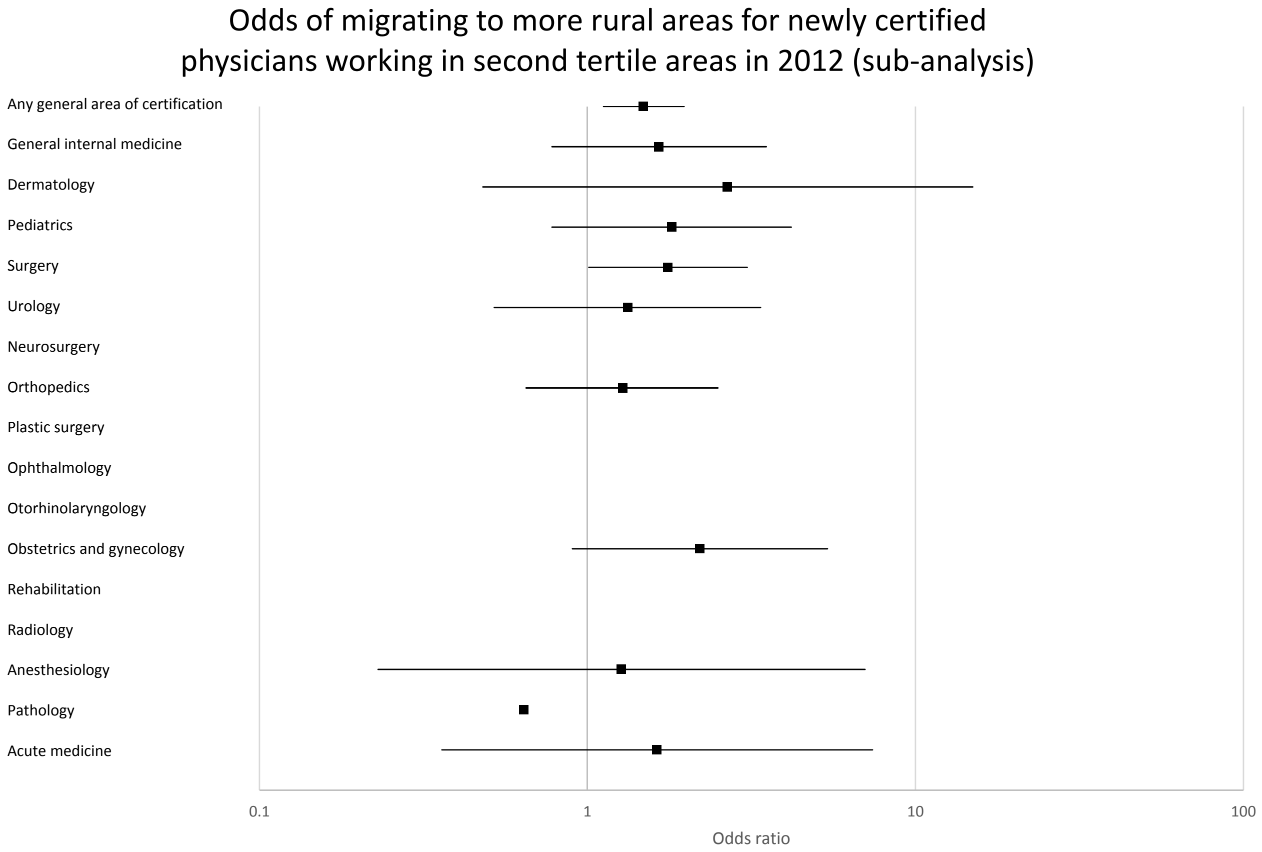

Odds of migrating to more rural areas for newly certified physicians working in second tertile areas in 2012 (sub-analysis)
Any general area of certification
General internal medicine
Dermatology
Pediatrics
Surgery
Urology
Neurosurgery
Orthopedics
Plastic surgery
Ophthalmology
Otorhinolaryngology
Obstetrics and gynecology
Rehabilitation
Radiology
Anesthesiology
Pathology
Acute medicine
0.1
1
10
100
Odds ratio


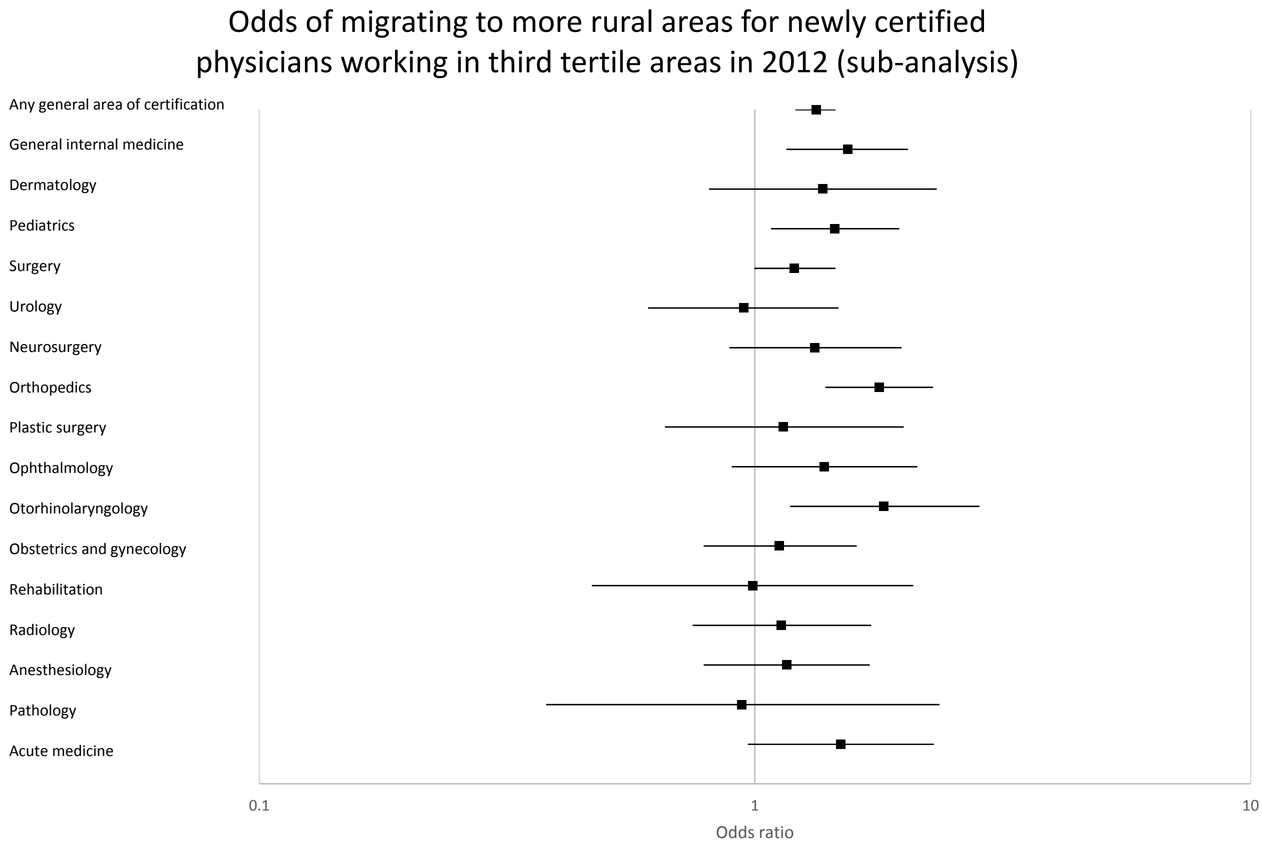

Odds of migrating to more rural areas for newly certified physicians working in third tertile areas in 2012 (sub-analysis)
Any general area of certification
General internal medicine
Dermatology
Pediatrics
Surgery
Urology
Neurosurgery
Orthopedics
Plastic surgery
Ophthalmology
Otorhinolaryngology
Obstetrics and gynecology
Rehabilitation
Radiology
Anesthesiology
Pathology
Acute medicine
0.1
1
10
Odds ratio
