# Supplementary figures and images for: Board certification and urban–rural migration of physicians in Japan
Source: BMC Health Serv Res. 2018 Aug 7;18:615. doi: 10.1186/s12913-018-3441-y (PMC6081900; doi:10.1186/s12913-018-3441-y)

## Rural practice and odds of keeping board certification (sub-analysis)

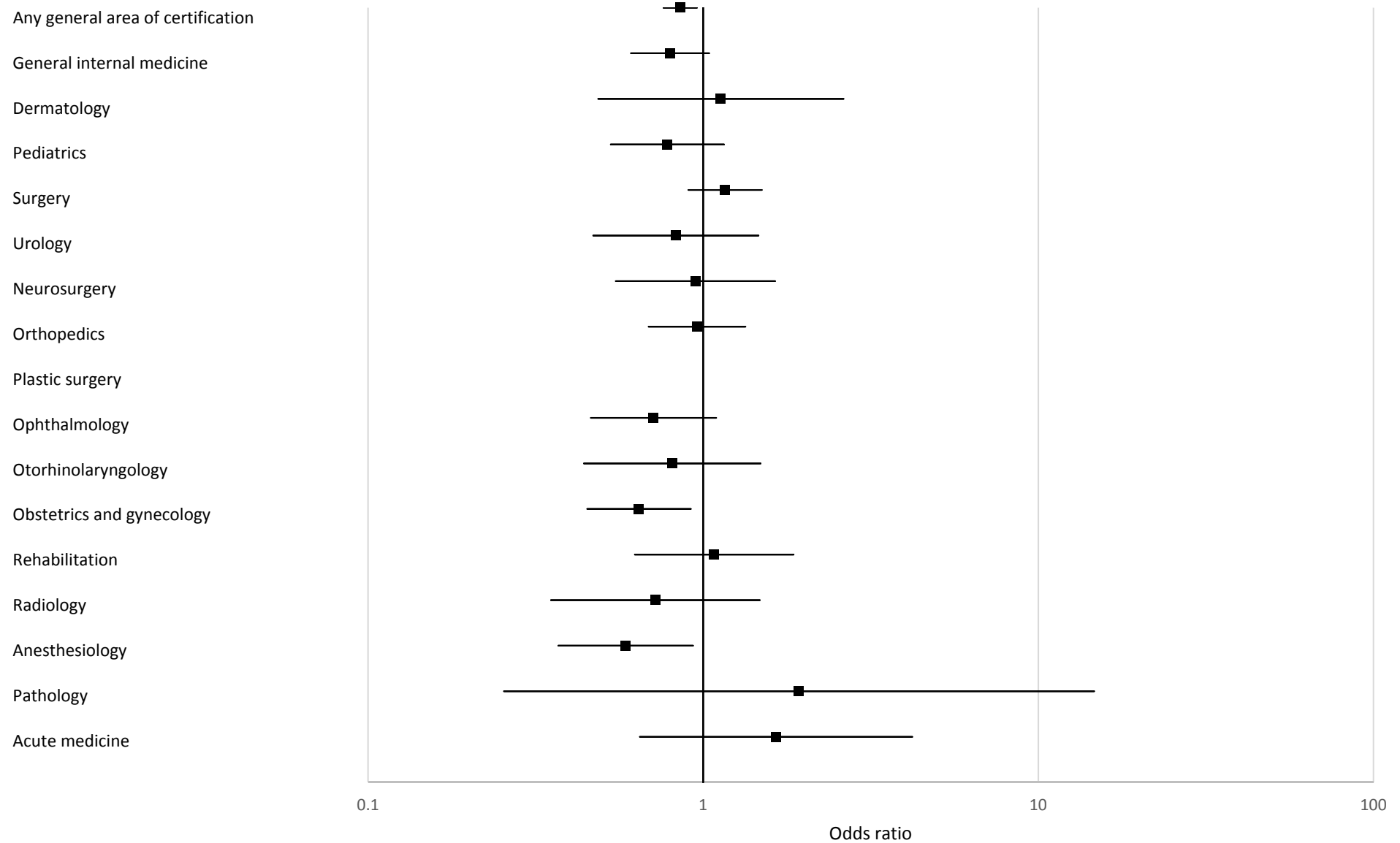

Supplement: Supplementary file 3 — Rural practice and odds of keeping board certification (sub-analysis of the individual specialties). (PDF 46 kb) [file 12913_2018_3441_MOESM3_ESM.pdf]
